# Solitary Langerhans cell histiocytosis of the hard palate: a diagnostic pitfall

**DOI:** 10.3205/000238

**Published:** 2016-09-19

**Authors:** Dalal Varsha, Manveen Kaur, Neena Chaudhary, Fouzia Siraj

**Affiliations:** 1 National Institute of Pathology, ICMR, New Delhi, India; 2 Department of ENT, Vardhman Mahavir Medical College and Hospital, New Delhi, India

**Keywords:** Langerhans cell histiocytosis, oral ulcer, immunohistochemistry

## Abstract

Langerhans cell histiocytosis (LCH) is a relatively rare and unique disease characterized by an abnormal proliferation of immature dendritic cells. It is predominantly seen in children with adults showing less than ten times the incidence compared to childhood. The clinical presentation and organ involvement is highly variable. Oral manifestations generally consist of mucosal ulceration associated with lesions of the underlying bone. Lesions limited to the oral mucosa are rare. We present a case of a 45-year-old male who presented with an ulcer on the hard palate showing histopathologic features of LCH. The present case is a reminder of the possibility of occurrence of this unusual entity in the oral cavity. Appropriate use of immunohistochemistry is advocated to avoid diagnostic pitfalls.

## Zusammenfassung

Langerhans-Zell-Histiozytose (LCH) ist eine relativ seltene und einzigartige Erkrankung, die durch abnorme Proliferation von unreifen dendritischen Zellen gekennzeichnet ist. Die LCH tritt überwiegend bei Kindern auf, bei Erwachsenen liegt die Häufigkeit bei 10% im Vergleich zur Inzidenz bei Kindern. Das klinische Bild und der Befall der Organe sind sehr variabel. Die Manifestation im Mundbereich besteht meist aus Schleimhautulzerationen verbunden mit Läsionen des darunterliegenden Knochens. Läsionen, die sich auf die Mundschleimhaut beschränken, sind relativ selten. Wir beschreiben den Fall eines 45 Jahre alten Mannes, der sich mit einem Geschwür am harten Gaumen vorstellte, das histopathologisch als LCH erkannt wurde. Der vorgestellte Fall soll daran erinnern, dass diese seltene Erkrankung in der Mundhöhle vorkommen kann. Geeignete immunhistochemische Verfahren werden empfohlen, um Fehldiagnosen zu vermeiden.

## Introduction

Langerhans cell histiocytosis (LCH) is a rare proliferative disorder of Langerhans cells of unknown etiology [1], [2]. It can involve multiple organ systems with varying presentations thus creating diagnostic dilemmas. The most frequently involved organs include bone, skin and lungs [2], [3]. Isolated oral lesions occur in about 5% of cases and are usually due to underlying bone involvement. Exclusive mucosal involvement in adults has rarely been reported with only a handful cases in literature [4], [5], [6], [7].

## Case description

**Figure 1:**
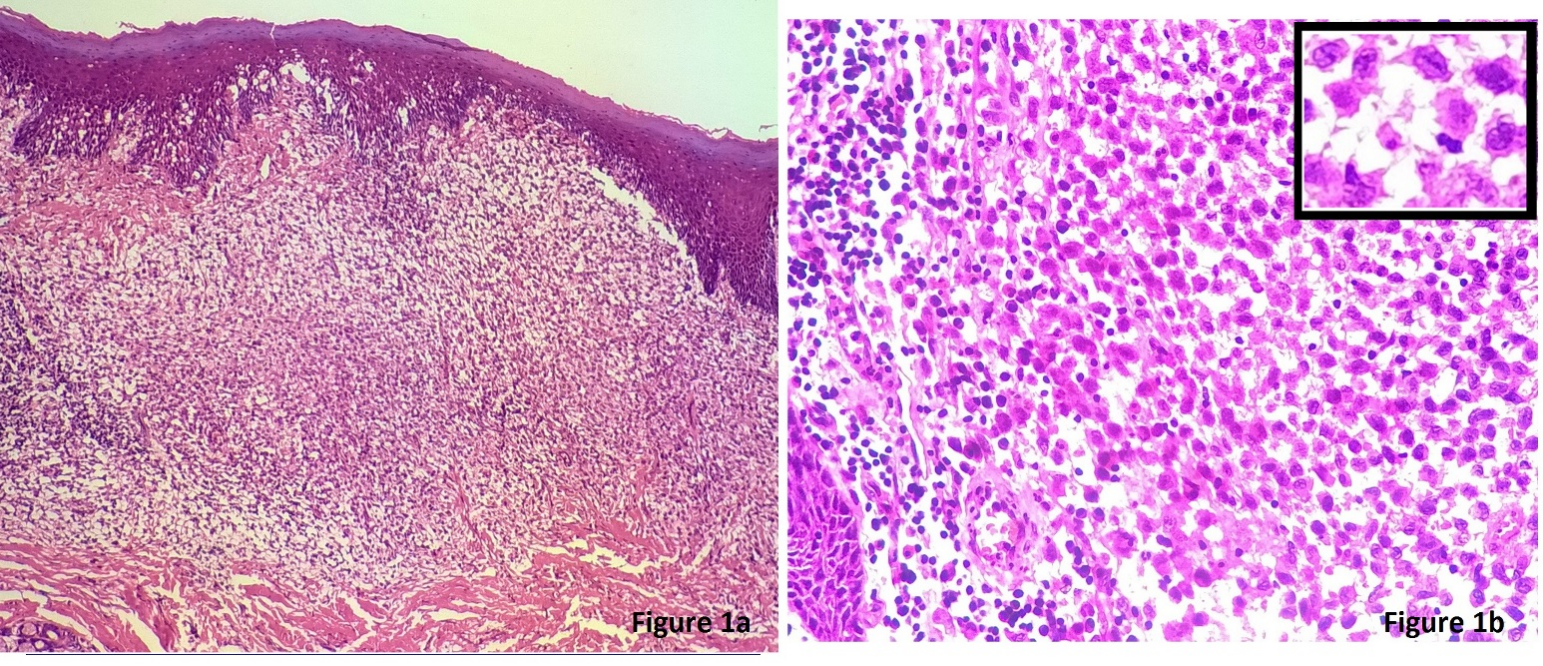
(a): Section showing oral mucosa and submucosa filled with dense infiltrate comprising inflammatory cells and sheets of large, polygonal cells. (b): Cells have moderate eosinophilic cytoplasm and reniform nuclei exhibiting grooves.

**Figure 2:**
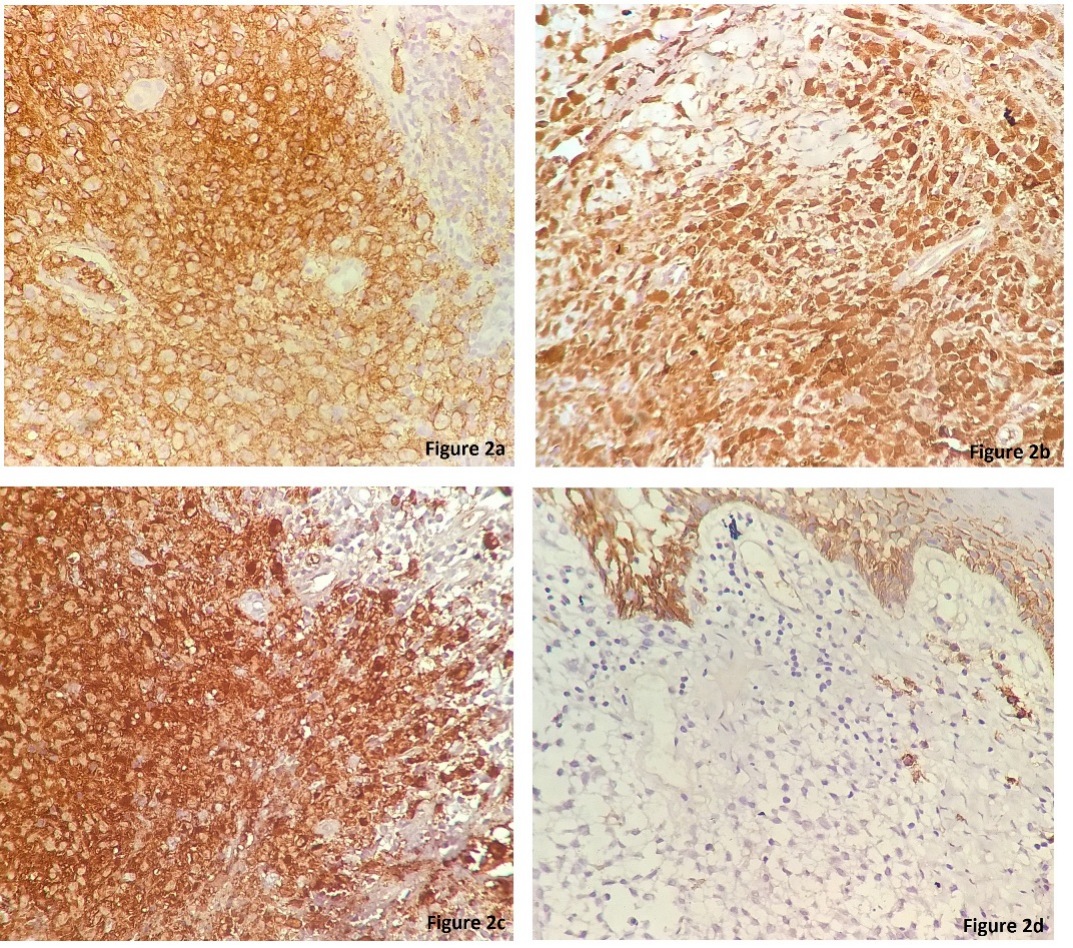
Immunohistochemical staining showing (a) LCA reactivity, (b) S100 reactivity, (c) CD1a reactivity in the large cells. (d) IHC staining for EMA is negative in these cells.

A 45-year-old male presented with an ulcer on the hard palate. Physical examination revealed an area of ulceration measuring 3x2 cm with ill-defined margins. X-ray did not show any bony lesion. On CT scan, no obvious mass lesion was observed in palate. Trucut biopsy from the site was reported elsewhere as poorly differentiated carcinoma, which was followed by right inferior maxillectomy. Gross examination of the specimen received showed an indurated area measuring 2.5x1.2x0.8 cm on the hard palate. Extensive sampling of this area revealed an ill-defined nodular lesion beneath the epithelium composed of sheets of large, polygonal cells with moderate eosinophilic cytoplasm and reniform nuclei exhibiting grooves (Figure 1 (Fig. 1)). These were admixed with lymphoid aggregates, plasma cells, eosinophils and neutrophils. On immunohistochemistry the large cells were diffusely positive for CD1a, S100, CD68 and negative for LCA, EMA, cytokeratin (Figure 2 (Fig. 2)). Sections from the underlying bone were unremarkable. Subsequent to the diagnosis, involvement of other systems was ruled out. The final diagnosis of Langerhans cell histiocytosis of the oral mucosa was rendered. Currently the patient is disease-free after a two year follow-up.

## Discussion

First described by Lichenstein in 1953, LCH is a disease of unknown cause [8]. Initially thought to be reactive, it has now evolved in its classification from a primary immune dysregulatory disorder to a dendritic cell neoplasm with an immune-inflammatory component. The disease encompasses a broad spectrum ranging from uni-focal organ manifestation with an excellent prognosis up to disseminated, progressive multi-organ involvement with a severe course and grim prognosis [2], [3], [6]. Three subtypes include eosinophilic granuloma, Letterer–Siwe disease, and Hand–Schüller–Christian disease according to solitary or multiple organ involvement [9]. 

LCH is predominantly a disease of childhood, incidence in adults being 10–15 per millions persons per year [3]. The organs most commonly involved in adults are the lungs, skeleton, and skin. Isolated oral lesions occur in about 5% of cases and are usually due to underlying bone involvement [3], [4], [6]. In oral soft tissue, the sites commonly involved are the gingiva followed by the maxillary and the hard palate [6]. Clinical presentation may mimic other conditions and can be in the form of hypermobile teeth, gingival hypertrophy, or ulcers of the mucosa, tongue, or lips as well as submucous nodules or leukoplakia [5], [9]. A complaint of oral ulcer in an elderly male may frequently raise the suspicion of malignancy and hence create a pitfall as was seen in the present case. 

Morphological examination in the present case revealed large polygonal cells in the subepithelium indicating a poorly differentiated tumor. However, close scrutiny showed nuclear grooves bringing histiocytic disorder in the differentials. A dense non-specific inflammatory infiltrate also hindered recognition of these tumor cells. According to The Working Group of the Histiocyte Society a definitive LCH diagnosis can be set when, in addition to these light microscopy features, Birbeck granules can be detected in lesional cells by electron microscopy, and/or a positive staining for CD1a antigen can be obtained [1], [2], [3]. Subsequently, immuoreactivity for S100 and CD1a in the tumor cellsboth in the biopsy and excised specimen confirmed the diagnosis of LCH.

Various treatment modalities for LCH have been proposed, including close observation, surgical curettage, local injection of corticosteroids, low-dose radiotherapy, high-dose systemic corticosteroids, chemotherapy and bone marrow transplantation and antibody therapy for resistant cases [4]. Recent studies documenting mutations in BRAF and MAP2K1 genes could revolutionize the prevalent therapeutic regimens and pave way for targeted therapy in LCH, resulting in a better prognosis [2]. In our case however, the patient underwent inferior maxillectomy on the basis of biopsy-proven squamous cell carcinoma. Presently, the patient is on close follow up and has been doing well for the past two years. 

Exclusive mucosal involvement without bone involvement by LCH, as in our case, is a rarity and often leads to delay in diagnosis. A clinical presentation of palatal ulcer in an elderly male often brings a differential diagnosis of malignancy in mind. However, careful search for classical Langerhans cells showing reniform nuclei with grooves and immunostaining for S100 and CD1a helps in reaching the final diagnosis. Our case reminds one to include LCH in the differential diagnosis of palate ulcers even in elderly patients.Also, since oral lesions may be the initial manifestations of a systemic involvement, histopathologic examination of these lesions becomes the cornerstone for further therapy and management. 

## Notes

### Competing interests

The authors declare that they have no competing interests.
